# Long noncoding RNA LOC100911498 is a novel regulator of neuropathic pain in rats

**DOI:** 10.1002/brb3.1966

**Published:** 2021-05-05

**Authors:** Wenxin Tang, Lufeng Zhang, Zhisong Li

**Affiliations:** ^1^ Department of Anaesthesiology The First Affiliated Hospital of Zhengzhou University Zhengzhou China

**Keywords:** brain‐derived neurotrophic factor, LOC100911498, microglia, neuropathic pain, P2X4R, p‐p38‐MAPK

## Abstract

**Introduction:**

Neuropathic pain (NP) is the most debilitating of all clinical pain syndromes and may be a consequence of dysfunction in the somatosensory nervous system. Unfortunately, the pathogenesis of NP is not fully understood yet and it cannot be cured totally. Long noncoding RNA (lncRNA) is a type of RNA molecule greater than 200 nucleotides, and dysregulated expression of lncRNAs play a critical role in the facilitation of NP. Previous study showed the expression level of LOC100911498 in the spinal cords of spared nerve injury (SNI) rats were increased. This research was aimed at exploring what role LOC100911498 plays in the pathophysiological process of NP.

**Methods:**

The mechanical withdrawal threshold (MWT) of rats was measured by the von Frey test. The expression levels of P2X4 receptor (P2X4R), ionized calcium‐binding adaptor molecule 1 (Iba‐1), p‐p38 and brain‐derived neurotrophic factor (BDNF) in spinal cords were detected, respectively.

**Results:**

Our results suggested that the level of LOC100911498 in SNI rats was markedly higher than that in the sham group; the MWT values in rats were treated with LOC100911498siRNA were increased, and the expression levels of P2X4R, Iba‐1, p‐p38 and BDNF in SNI+ LOC100911498siRNA group were reduced compared with those in the SNI group.

**Conclusion:**

Our study indicated the effects lncRNA LOC100911498 siRNA exerted on NP were mediated by P2X4R on microglia in the spinal cords of rats. Further, LOC100911498 may be a novel positive regulator of NP by regulating the expression and function of the P2X4R.

## Graphical abstract

LOC100911498 might participate in the development of neuropathic pain. LOC100911498 siRNA treatment could relieve the pain. LOC100911498 siRNA could block P2X4Rs‐mediated p38MAPK activation and BDNF release.

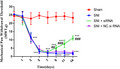

## 1 INTRODUCTION

Neuropathic pain (NP) is a debilitating pain condition directly caused by injury or disease of the somatosensory nervous system and a serious condition that can cause a series of symptoms, such as allodynia, hyperalgesia, and spontaneous pain (Finnerup et al., 2016). Although great efforts have been to cure the problem, advances in molecular mechanisms and therapies for it have hitherto been limited. Thus, NP is widely considered as one of the most difficult pain syndromes to manage and its unsatisfactory outcomes can lead to severe socioeconomic impacts (Gilron et al., 2015; Van Hecke et al., 2014).

Long noncoding RNAs (lncRNAs) are a type of transcripts greater than 200 nucleotides that lack important protein‐coding capacity. Emerging data have revealed that lncRNAs are associated with various biological processes, including dosage compensation and genomic imprinting, embryo development and cell differentiation, the development of genetic disorders, etc (Melissari & Grote, 2016). Recent studies have suggested lncRNAs play a crucial role in the formation of NP via upregulation or downregulation in NP models (Jiang et al., 2015; Lalevee & Feil, 2015; Li et al., 2017; Liu et al., 2016; Zhou, Fan et al., 2017; Zhou, Xiong et al., 2017). It has attracted much attention in aberrant expression of lncRNAs in NP and supports potential role of lncRNAs as new therapeutic targets for the treatment of NP.

Adenosine‐5′‐triphosphate (ATP) is associated with the analgesia transmission, and previous evidence demonstrated extracellular ATP involves the occurrence of mechanical hyperalgesia in spared nerve injury (SNI) rats by activation of purinoceptors (Burnstock, 2016; Puchalowicz et al., 2014, 2015). ATP can activate ionotropic receptor P2X (P2X_1–7_) and G‐protein‐linked receptor P2Y (Burnstock, 2006, 2007, 2009). P2X4 is one of the most sensitive purinergic receptors, it is about one thousand times higher than the archetypal P2X7 at nanomolar ATP concentrations (Suurvali et al., 2017). A growing body of evidence indicates that microglial P2X4 receptors (P2X4Rs) make a critical contribution to the mechanisms underlying NP (Beggs et al., 2012; Inoue & Tsuda, 2012; Tsuda et al., 2013). And the levels of P2X4 protein and messenger RNA (mRNA) in the spinal cords are upregulated following SNI (Zhou et al., 2014). Consequently, microglia P2X4Rs are defined as a suitable target for treating NP.

The LOC100911498 is lncRNA (3233927472979998363‐8.pdf). It has been shown to be elevated in the rat spinal cords in response to SNI (Zhou, Xiong, et al., 2017), suggesting that lncRNA LOC100911498 might be a key factor in the transmission of nociceptive signaling. The aim of our study was to explore the effects of lncRNA LOC100911498 small interference RNA (siRNA) on P2X4 receptor‐mediated NP.

## 2 EXPERIMENTAL PROCEDURES

### 2.1 Animals and groups

Healthy adult male Sprague Dawley (SD) rats (weight 180–220 g) were provided by the Laboratory Animal Center of Zhengzhou University, Zhengzhou, Henan Province, People's Republic of China. They were housed under constant conditions of temperature (22 ± 2°C), relative humidity (60% ± 70%), and a 12‐hr artificial light/dark cycle (lights on at 8 a.m.), with standard laboratory rat chow and tap water ad libitum. All experiments were carried out in accordance with protocols approved by the National Institutes of Health on animal care and the ethical guidelines for the experimental investigation (Zimmermann, 1983). To test the effects of LOC100911498 siRNA, rats were randomly assigned to 4 groups: (1) sham control group (Sham); (2) SNI group (SNI); (3) SNI rats transfected with LOC100911498 siRNA group (SNI+ LOC100911498 siRNA); and (4) SNI rats transfected with scramble siRNA group (SNI + NC siRNA).

### 2.2 Intrathecal catheter implantation and drugs delivery

Intrathecal polyethylene‐10 (PE‐10) catheters were implanted into all the rats according to the method of Storkson et al., (1996). Reduce mortality and infection rates of animals with modifications. Rats were anesthetized with sevoflurane (2%–3%) vaporized through a nose cone during surgical procedures, making a skin incision in midline lumbar region (L4–S1). Each polyethylene catheter (PE‐10, 18 ± 2 cm) was inserted into the subarachnoid space rostrally at the lumbar enlargement through a hole between L4 and S1 and fixed at the posterior nuchal area to the top of the occipital region. The rats were allowed to recover for a minimum of 7 days before the actual experiment. A motor behavioral testing is essential before experiments, and rats with motor deficits were excluded from study. The siRNA specific for LOC100911498 and scramble siRNA were purchased from GenePharma (Shanghai, China) and dissolved in RNA‐free water prior to use. From the seventh day after surgery, LOC100911498 siRNA or vehicle was injected at a dose of 1 OD siRNA/20 µl/rat once daily for 5 consecutive days. The siRNA sequence selected for LOC100911498 was CCGAGUAAUUUGACGUAUUTT.

### 2.3 SNI

To generate the SNI model (Decosterd & Woolf, 2000), each rat was anesthetized with sevoflurane (2%–3%) vaporized through a nose cone during surgical procedures. The femur at the left mid‐thigh level was used as the incision landmark, and the skin on the lateral surface of the thigh was incised. Separate the biceps femoris muscle and reveal the sciatic nerve and its three terminal branches: the sural, common peroneal, and tibial nerves. The common peroneal and the tibial nerves were tight‐ligated with 5.0 silk and sectioned distal to the ligation, removing 2 ± 4 mm of the distal nerve stump. Left sural nerve was preserved carefully to avoid any contact with surgical instruments or nerve stretch, it was remained intact. Closed muscle and skin in 2 layers with 4.0 silk. The sham group underwent exactly same procedures except nerve ligation and transection.

### 2.4 Mechanical paw withdrawal

The mechanical paw withdrawal threshold (MWT) was mearsured as applying mechanical stimuli to the right hind paw responded to von‐Frey hairs, and 50% MWT was determined using the up–down method (Chaplan et al., 1994). The test area was the lateral part of the plantar surface of the paw (sural nerve skin territory). All rats were acclimated to the testing environment for at least 30 min before measurements, and the interstimulus interval was 5 min. Held in place for approximately 6–8 s when alogarithmic series of von‐Frey hairs (0.4–15.1 g) were presented perpendicularly to the plantar surface in ascending order of strength. A positive response was defined if the hind paw withdrawn or licking of the paw immediately upon the stimulus presentation. MWT was executed on −1, 1, 3, 5, 7, 9, 11, 14 days after surgery in a blinded manner.

### 2.5 Real‐Time PCR

Ipsilateral lumbar 4–5 (L_4–5_) spinal dorsal horn tissues of rats were isolated and stored in −80°C refrigerator temporarily on the 14th day after surgery. Total RNA was extracted using the TRIzol method (TransGen Biothch, Beijing, China). Total RNA (2 μg) was converted to cDNA using Biometra TAdvanced (Analytik Jena AG, Jena, Germany). The primers were designed and purchased from Invitrogen (Thermo Fisher Scientific, CA, USA), and the sequences were as follows: β‐actin, forward 5′‐TAAAGACCTCTATGCCAACACAGT‐3′ and reverse 5′‐CACGATGGAGGGGCCGGACTCATC‐3′; P2X4, forward 5′‐AGACACTGCTGTGGCTTAGG‐3′ and reverse 5′‐GCTGACAGCACCTGAGAGAG‐3′; LOC100911498 forward 5′‐TGTTGGGACAGCTTTATCAG‐3′ and reverse 5′‐GCAGGTAGCCTTCATTTGG‐3′. PCR was performed with the SYBR Premix Ex Taq II (Takara Bio Inc., Kusatsu, Japan) in an ABI‐Prism 7500 Sequence Detection System (Applied Biosystems, CA, USA). Relative concentrations of mRNAs were calculated on the basis of threshold cycle values and corrected by β‐actin expression. The result was measured following to the comparative 2^−△△CT^ method.

### 2.6 Western blot analysis

Ipsilateral spinal cord tissues were collected and homogenized in 1,000 µl ice‐cold cytoplasmic lysis of RIPA Lysis Buffer (Beyotime Biotechnology, Shanghai, CN) and phosphatase inhibitor cocktail (CWBIO, Beijing, CN). Then, the homogenate sample was centrifuged at 12,000 rpm for 15 min at 4°C and collected the supernatant containing membrane protein samples. Protein samples were dissolved in loading buffer and denatured at 99°C for 5 min. 30–60 μg protein was subjected to 8%–12% SDS polyacrylamide gel electrophoresis and transferred onto a polyvinylidene difluoride membranes (PVDF, Millipore, Bedford, MA, USA). The PVDF membranes were blocked in 5% skim milk for 2 hr and then incubated overnight at 4°C with the following primary antibodies (rabbit anti‐P2X4Rs, 1:200, Alomone Labs; rabbit anti‐BDNF, 1:1,000, Abcam; rabbit anti‐p38, Abclonal; rabbit anti‐p‐p38, Abclonal and rabbit anti‐GAPDH, 1:500, Goodhere Biotechnology Co.). The proteins were detected with a HRP‐conjugated goat anti‐rabbit secondary antibody IgG (1:5,000, Beijing Zhongshan Biotech Co.). Specific bands of target proteins were visualized using the chemiluminescence reagents provided with the ECL kit (Solarbio, Beijing, CN) and exposed to film. The band densities were determined using Image J Software and normalized to each internal control.

### 2.7 Immunohistochemistry

On the 14th day after surgery, the animals were deeply anesthetized with penthiobarbital sodium and transcardially perfused from the ascending aorta with normal saline and 4% paraformaldehyde (PFA) in 0.1 mol/L phosphate buffer (pH 7.4), respectively. After L_4–5_ spinal cords were removed, post fixed in same fixative for 3 hr, and dehydrated by 30% sucrose in phosphate buffer over two nights. After being cut into 25‐μm thick sections using a cryostat, the transverse spinal sections were processed for immunofluorescence. All the blocked sections were blocked with 5% goat serum in 0.3% Triton X‐100 for 1 hr at room temperature (RT) and incubated with mouse anti‐iba1 (1:500, Abcam) and rabbit anti‐P2X4Rs (1:200, Alomone Labs) over night at 4°C. After three rinses in PBS, all the sections were treated by a mixture of Alexa 647 donkey anti‐mouse IgG and Alexa 488 donkey anti‐rabbit IgG (1:200, Thermo Fisher) for 2 hr at RT, and thereafter, the preparations were washed in PBS. Olympus IX73 (Olympus Optical, Olympus Co. Ltd., Tokyo, Japan) fluorescence microscope was used to analyzed the stained sections, and images were captured with a CCD spot camera. The primary antibodies were omitted during staining when serving as a negative control, and further examined by Western blot.

### 2.8 Predicting the interaction between LOC100911498 RNA and P2X4 receptors

It is well known that there are interactions between RNA and proteins (Nacher & Araki, 2010). We took advantage of the application of bioinformatics technology (CatRAPID) to predict these interactions rapidly to evaluate the interaction of the LOC100911498 RNA and P2X4R, on the basis of secondary structure, hydrogen bonding, and molecular interatomic forces (Bellucci et al., 2011). The interaction propensity implies interaction probability between target RNA and target protein, and the discriminative power represents the confidence of the interaction propensity. The CatRAPID result demonstrated a high score for the interaction between LOC100911498 and P2X4R.

### 2.9 Statistical analysis

Statistical tests were performed with SPSS 10.0 (SPSS Inc., USA) and SigmaStat (Systat, San Jose, CA). All data are expressed as mean ± standard error. For MWT, the data were analyzed by two‐way (time and treatment) repeated measures analysis of variance (ANOVA) followed by Newman–Keuls post hoc test and *t* test between 2 groups at the same time points was carried out. One‐way ANOVA was used to test for statistical differences of Western blot, Immunofluorescence and real‐time PCR data followed by Tukey's or Dunnett's tests, while ISH was tested by *t* test as only 2 groups were applied. *p* < .05 was considered statistically significant.

## 3 RESULTS

### 3.1 Changes in LOC100911498 expression in SNI rats

**Figure 1:**
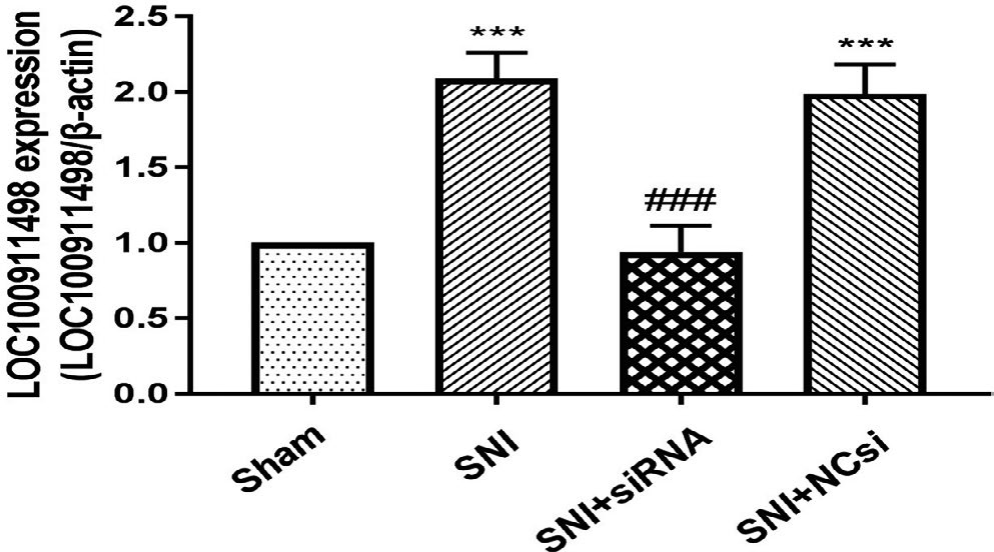
Changes in LOC100911498 expression in SNI. The expression of LOC100911498 in the spinal cords by real‐time PCR. The expression of LOC100911498 in the SNI group was increased compared with the control group. Treatment of the SNI group with LOC100911498 siRNA decreased the expression of LOC100911498 compared with the SNI rats. Mean ± *SEM*, *n* = 6, ***p < .001 compare with the control group, ^###^ *p* < .001 compare with the SNI group

Real‐time PCR presented that compared with the control group, SNI also causes upregulation in the LOC100911498 expression of the spinal cords (*p* < .001), whereas intrathecal transfection of LOC100911498 siRNA prevents over‐expression of LOC100911498 (*p* < .001). And no difference in the LOC100911498 expression of spinal cords was found between the SNI and SNI + NCsi groups (*p* > .05; Figure 1).

### 3.2 Effects of LOC100911498 siRNA on mechanical hyperalgesia in SNI rats

**Figure 2:**
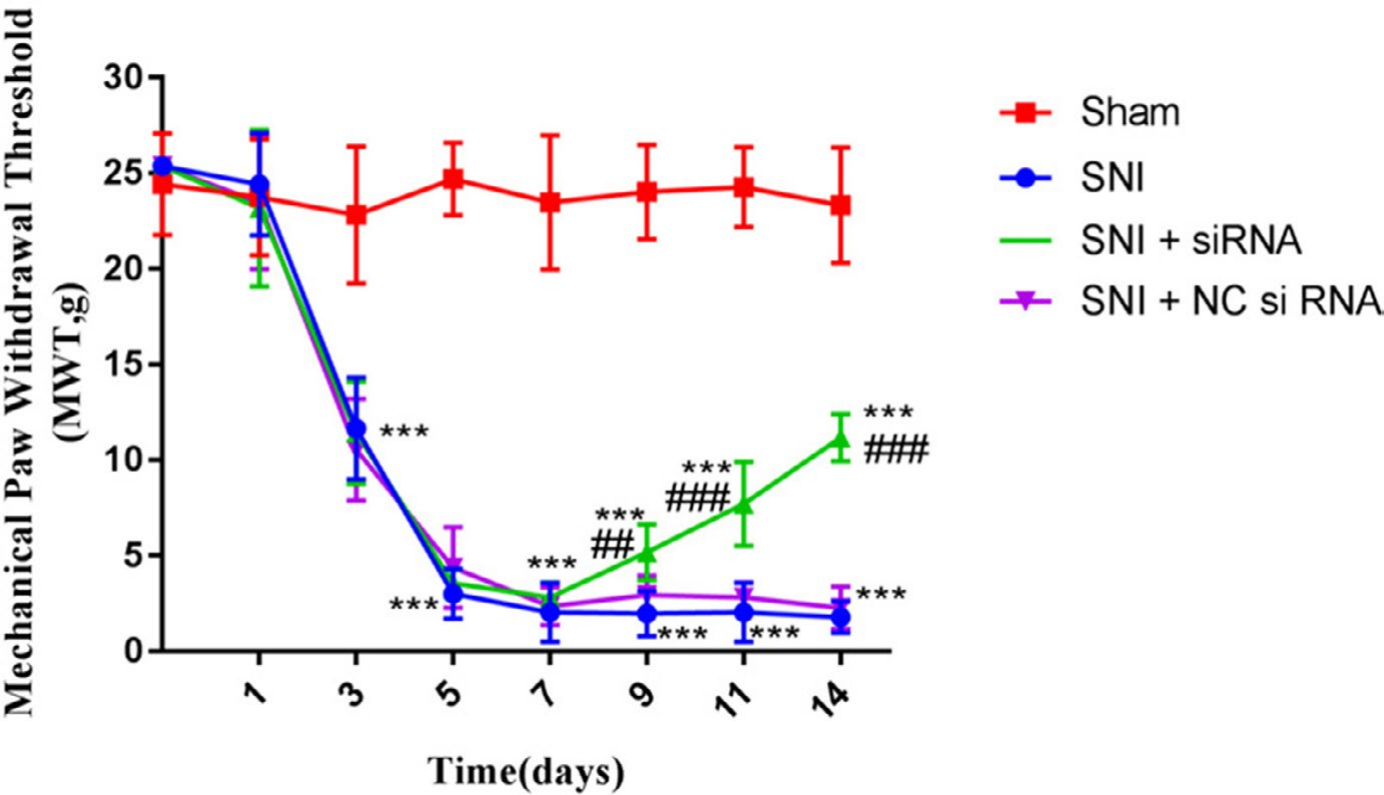
Effects of LOC100911498 siRNA in the mechanical withdrawal threshold (MWT) in SNI rats. The MWT in SNI rats treated with LOC100911498 siRNA was higher than that in the SNI group. There was no difference between the SNI group and SNI + NCsiRNA group. The upper limit of MWT detection was 25.368 g. Mean ± *SEM*, *n* = 8, ***p < .001 compare with the control group, ^##^ *p* < .01, ^###^ *p* < .001 compare with the SNI group

Compared with the control group, MWT in SNI group was considerably elevated (*p* < .001). In contrast, MWT in SNI + LOC100911498si group was decreased in comparison with SNI and SNI + NC siRNA groups, (*p* < .001), but still lower versus the sham group (*p* < .001). There was no significant difference in MWT between the SNI and SNI + NC siRNA groups (*p* > .05; Figure 2). Combined with the previous result, we could infer that LOC100911498 may be involved in the generation of mechanical allodynia induced by SNI.

### 3.3 Predicting the interaction between LOC100911498 RNA and P2X4Rs

**Figure 3:**
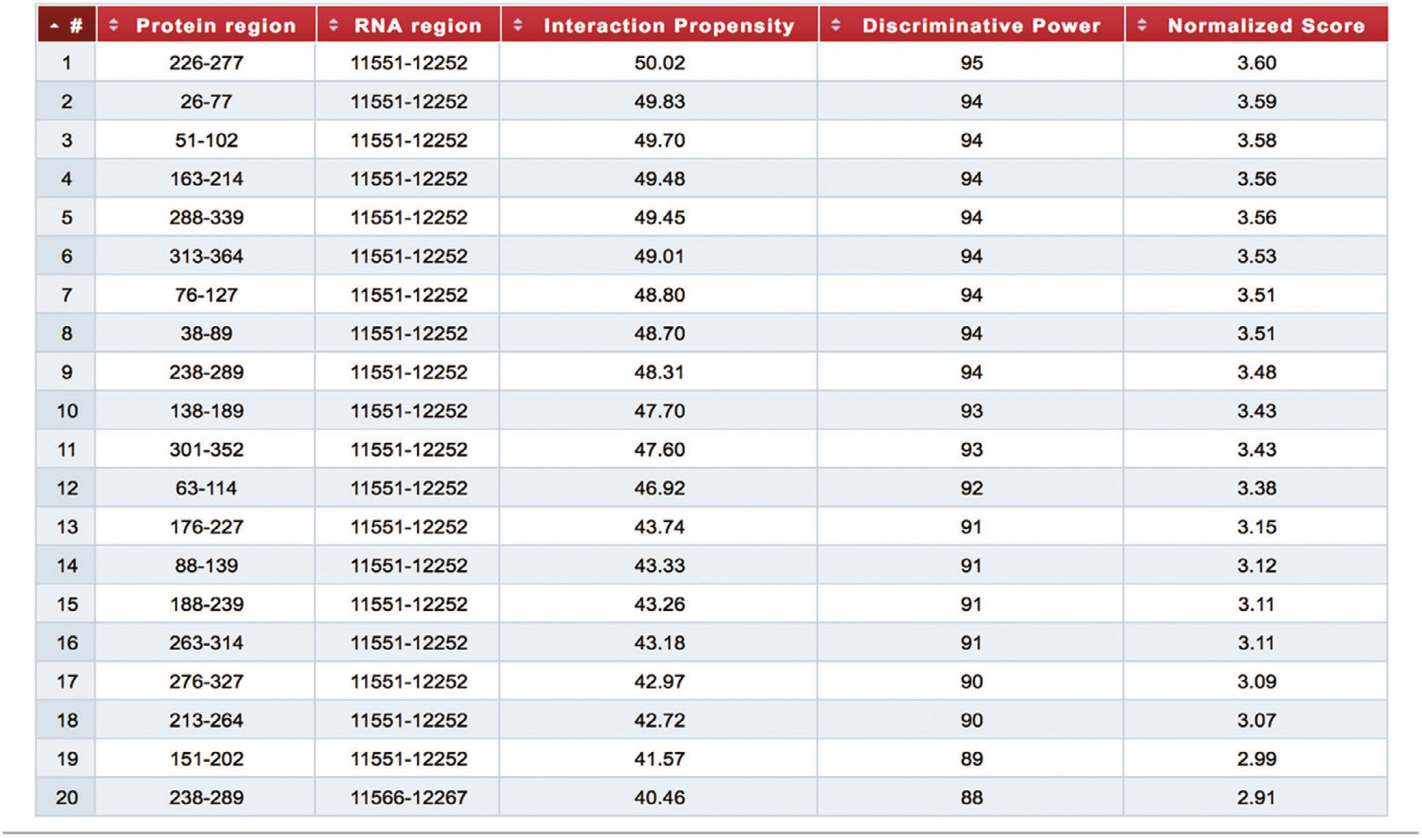
The catRAPID identified the interaction between LOC100911498 and P2X4 receptor. The actual prediction result of LOC100911498 and P2X4 was shown as a table. The result table summarizes the top 20 interactions, reporting the coordinates of both P2X4 and LOC100911498 RNA fragments that have been employed in the analysis, their interaction propensity, the discriminative power of each interaction and the normalized score used for the graphical representations

An important mechanism underlying LOC100911498 regulation of NP is through interactions with G‐protein‐coupled receptors and ion channels, which are primarily participated in the initiation and maintenance of NP (Julius & Basbaum, 2001; Tsuda et al., 2013; Veldhuis et al., 2015). One of the most possible candidates is the P2X4Rs that have been considered implicated in NP after peripheral nerve injury (Beggs et al., 2012; Inoue & Tsuda, 2012; Puchalowicz et al., 2014; Suurvali et al., 2017; Trang & BEGGS S et al., 2009; Tsuda et al., 2013). Thence, inhibiting ATP activation of P2X4Rs is a new approach to solve NP. And protein‐RNA interactions can be predicted and evaluated via an online algorithm called CatRAPID. CatRAPID provides a method to perform large‐scale predictions of protein‐RNA associations based on their sequences, secondary structures propensities, hydrogen bonding, and van der Waals (Bellucci et al., 2011). So we used CatRAPID to identify potential interaction between LOC100911498 and P2X4Rs. Of interested, the result given revealed that LOC100911498 has a strong correlation with P2X4Rs. It means the interaction is likely to exist (Figure 3).

### 3.4 Effects of LOC100911498 siRNA on the expression levels of P2X4Rs mRNA or protein in the spinal cords

**Figure 4:**
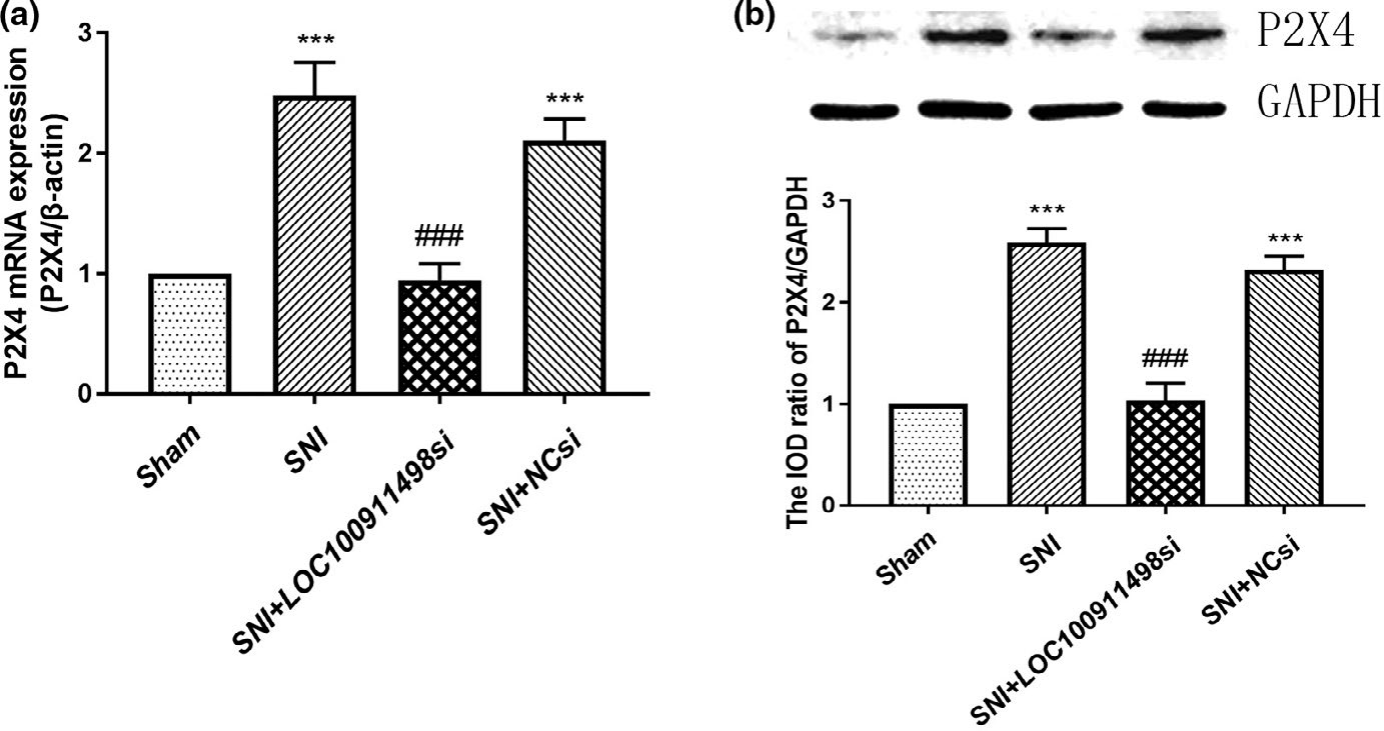
Effects of LOC100911498 siRNA on P2X4 mRNA and protein expression levels in the spinal cords of SNI rats. The integrated optical density values of the P2X4 mRNA (a) and protein (b) expressions in the SNI group were much higher compared with the control group. The expression levels of the P2X4 mRNA and protein in the SNI + NCsiRNA group were decreased compared with SNI rats. *n* = 6, ***p < .001 versus sham group, ^###^ *p* < .001 versus SNI group

The expression of spinal cords P2X4 mRNAs in each group was evaluated using real‐time PCR. The result showed apparently upregulated expression of P2X4Rs mRNA in the spinal dorsal horn of SNI rats versus the sham control group (*p* < .001). While the P2X4Rs mRNA expression in SNI rats injected with LOC100911498 siRNA was reduced compared with rats in the SNI group (*p* < .001), P2X4Rs mRNA expression of spinal cords between the SNI and SNI + NC siRNA groups did not differ (*p* > .05; Figure 4a).

Consistent with the PCR results, quantitative analyses of Western blot indicated that the integrated optical density (IOD) values of P2X4Rs protein expression in the SNI rats were obviously higher at day 14 after SNI than those in the sham rats (*p* < .001). And P2X4Rs protein expression levels in the SNI + LOC100911498 siRNA group were lower compared with those in the SNI group (*p* < .001). Similarly, no statistical significance in the P2X4Rs protein expression of spinal cords appeared between the SNI and SNI + NC siRNA groups (*p* > .05). These results suggested that LOC100911498 siRNA treatment could influence the function of P2X4Rs in SNI rats, and tonic activation of P2X4Rs in microglia might account for sustaining allodynia (Figure 4b).

### 3.5 Effects of LOC100911498 siRNA on the coexpression of P2X4Rs and iba‐1 in spinal cords based on immunofluorescence

**Figure 5:**
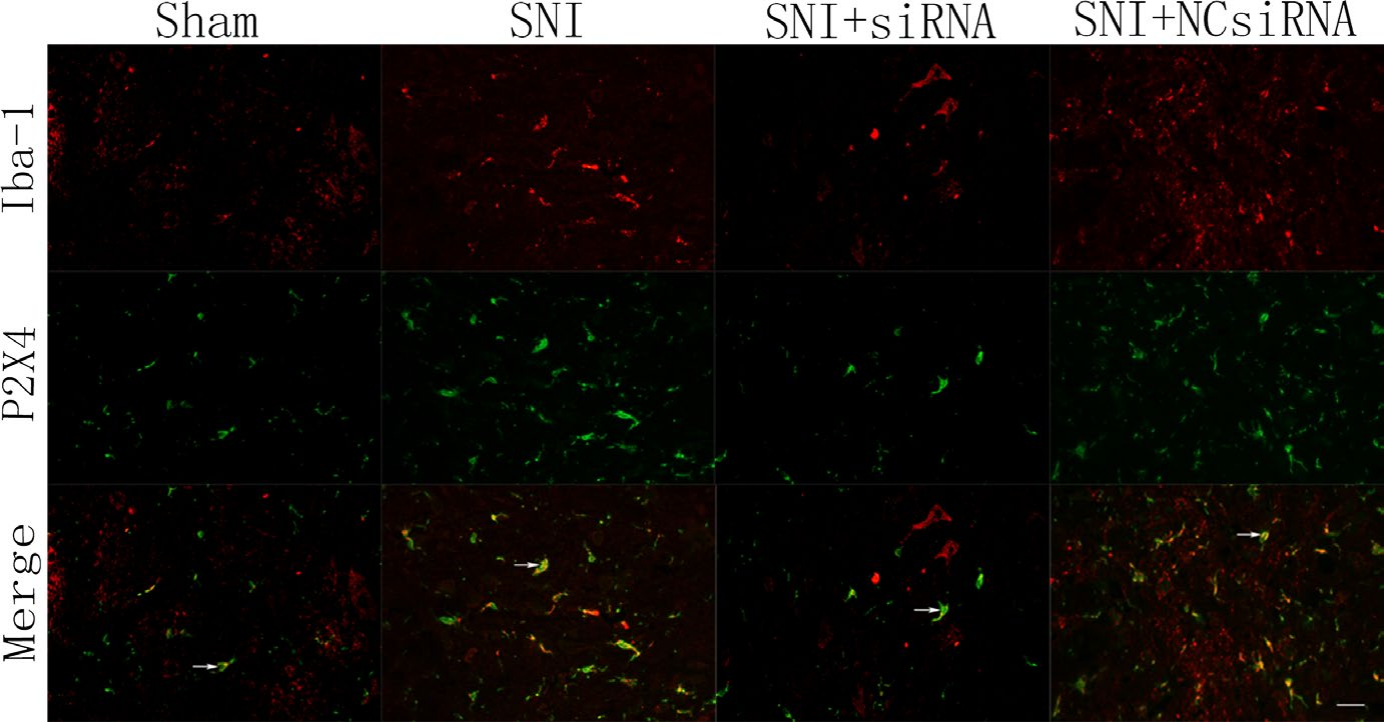
Effects of LOC100911498 siRNA on the expression levels of dorsal horn immunofluorescence in SNI rats. The coexpression quantities of Iba‐1 and P2X4 were increased in the spinal cords of SNI rats. The coexpression values of the P2X4Rs with Iba‐1 in SNI rats were higher than those in the control group. The red signal represents Iba‐1 staining with TRITC (tetraethyl rhodamine isothiocyanate), the green signal indicates P2X4 staining with FITC (fluorescein isothiocyanate), and merge represents the Iba‐1 and P2X4 double staining image. Scale bar: 20 μm

Ionized calcium‐binding adaptor molecule 1 (Iba‐1) is the marker of microglial cells. Immunoluorescence technique was used to examine the coexpression of P2X4Rs and Iba‐1 in spinal dorsal horn. Representative immunofluorescence further confirmed the above results. As shown by double immunofluorescence staining, the fluorescent Iba‐1 labels were consistently distributed with the P2X4Rs label (Figure 5). The coexpression of the P2X4Rs with Iba‐1 is much stronger in the spinal cords of SNI rats than that in the control group. It meant microglial cells are activated and P2X4Rs expression increases in the case of peripheral nerve injury stimulus. On the contrary, injection of LOC100911498 siRNA remarkably decreased both Iba‐1 and P2X4Rs immunoreactivity as well as the number of Iba‐1/P2X4Rs double‐positive cells, whereas no such changes were observed in the SNI + NCsi group, and there was no apparent difference between the sham and SNI + NCsi groups. In accordance with these data, we hypothesized it is possible that LOC100911498 siRNA knockdown reduces the increase of P2X4Rs related to microglial cells activation in the spinal dorsal horn.

### 3.6 Effects of LOC100911498 siRNA on p‐p38 and brain‐derived neurotrophic factor (BDNF) in the spinal cords

**Figure 6:**
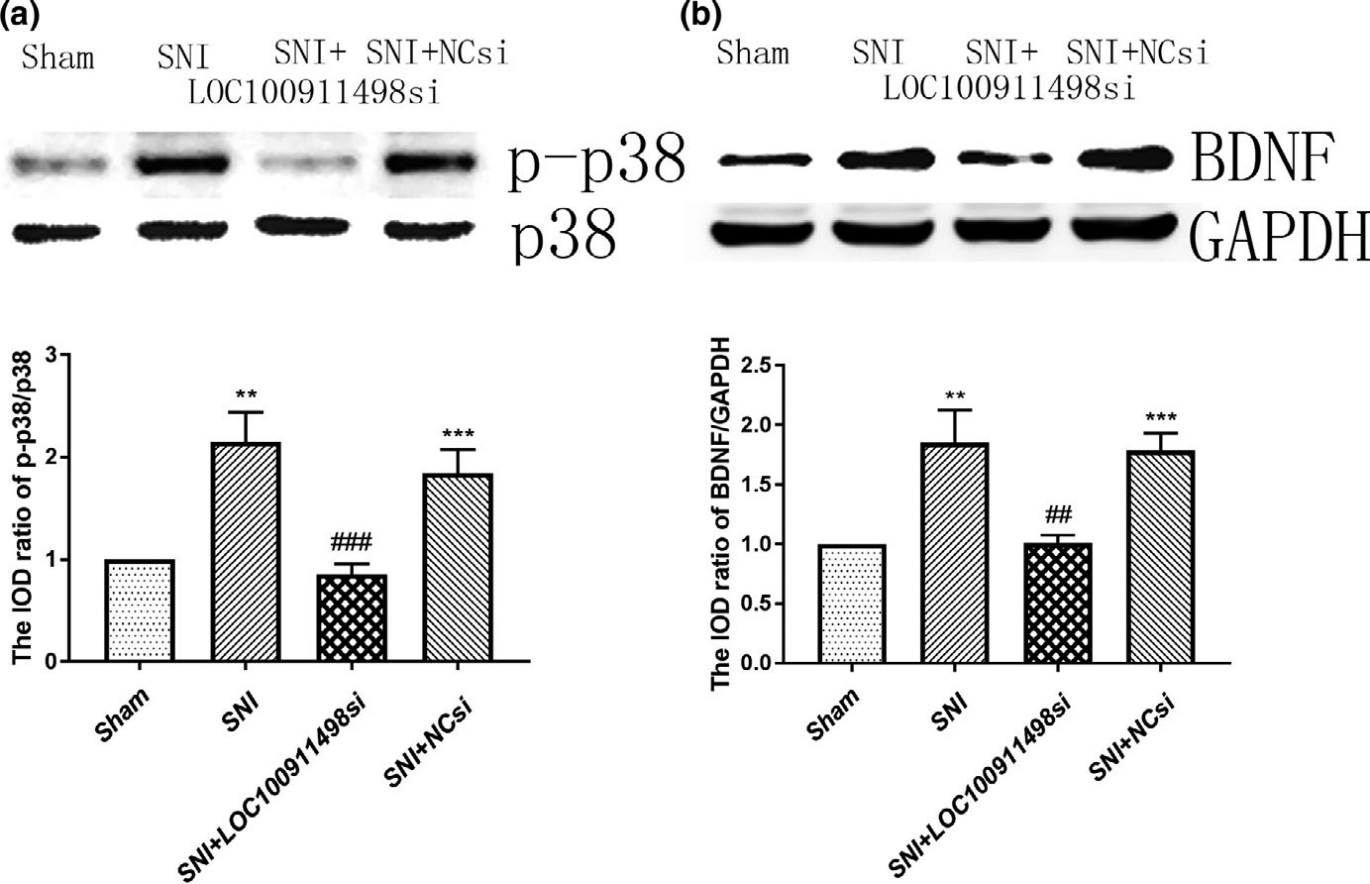
Effects of LOC100911498 siRNA on p‐p38 and brain‐derived neurotrophic factor (BDNF) expression of SNI rats. The expressions of p‐p38 (a) and BDNF (b) in the SNI group suggested a significant increase in comparison with the control group. The SNI + LOC100911498si group demonstrated a decrease in the levels of pp38 and BDNF compared with the SNI group. *n* = 6, *p < .05, **p < .01, ***p < .001 versus sham group, ^##^ *p* < .01, ^###^ *p* < .001 versus SNI group

A candidate signaling molecule implicated in initiation and maintenance of NP after peripheral nerve injury is the mitogen‐activated protein kinase (MAPK) family member, p38‐MAPK Jin et al., 2003; Tsuda et al., 2004; Zhuang et al., 2007). And P2X4Rs stimulation‐induced phosphorylation of p38‐MAPK appears necessary for both the release and synthesis of BDNF (Trang & BEGGS S et al., 2009), which participated in NP progression. The expression values of phospho‐p38 (p‐p38) and BDNF in spinal cords were measured by Western blotting. The IOD ratio of p38 to GAPDH was not significantly different among the four groups (*p* > .05). Nevertheless, the IOD ratio of p‐p38 to p38 in the SNI group was remarkably upregulated compared with that in the control group (*p* < .01; Figure 6a). Similarly, in comparison with the control rats, the IOD ratio of BDNF to GAPDH in the rats treated with SNI was increased (*p* < .01; Figure 6b). These results revealed that the roles of p38MAPK phosphorylation and BDNF in the spinal cords were associated with the P2X4Rs‐mediated hyperalgesia in SNI rats.

As expected, intrathecal treatment with LOC100911498 siRNA exerted considerable effects on preventing increased p‐p38 and BDNF protein expressions. The concentrations of p‐p38 and BDNF in the SNI rats transfected with siRNA were much lower than those in the SNI group (*p* < .001; Figure 6a,b). No statistical differences were observed between the SNI and SNI + NC siRNA groups (*p* > .05). Taken together, our findings suggested that LOC100911498 siRNA treatment might be responsible for the decrease of the phosphorylation of p38MAPK and the expression of BDNF in the spinal cords of SNI rats to alleviate P2X4Rs‐mediated pain hypersensitivity.

## 4 DISCUSSION

Neuropathic pain is a difficult type of chronic pain, the exact causes, pathophysiology, and mechanism remain vague, and the conventional therapy of NP is still unsatisfactory. Recently, increasing studies have observed the important cellular functions of lncRNAs to contribute to the pathogenesis of NP (Dou et al., 2017; Li et al., 2017; Liu et al., 2016; Peng & Zou et al., 2017). A lot of lncRNAs have been fully identified in the spinal cord dorsal horn, dorsal root ganglion (DRG), and the injured peripheral nerves from various rodent models NP and in the serum from NP patients. The lncRNAs are dysregulated in these pain‐related regions. In addition, the research reveals that lncRNAs play a contributory role in NP by underlying mechanisms. LncRNAs may directly bind to the target proteins and potentiate the pain‐related currents. Alternatively, lncRNAs might associate with pain‐associated molecules and downstream signals through regulating the gene expressions (such as sponging mRNAs or interacting with circRNA). Therefore, these studies have heightened interest in the possibility that the lncRNAs could be a potent experiment direction for the progression of NP therapy.

However, the field regarding the role of lncRNAs in the development and maintenance of NP is still in its infancy compared to tumor research. The detailed mechanisms of how these lncRNAs regulate the gene and protein expressions and interact with a large number of noncoding RNAs are still elusive. And the functions of most dysregulated lncRNAs identified by RNA sequencing in NP are still vague. Among them, the role of lncRNA LOC100911498 in NP has not been reported.

The SNI model is regarded as long‐time mechanical allodynia, which is NP symptom. In SNI rats, the common peroneal and tibial nerves are injured, generating continuous and reproducible pain hypersensitivity in the area of the spared sural nerve (Bourquin et al., 2006). In our study, the results of RT‐PCR and in situ hybridization have revealed that lncRNA LOC100911498 might be positively related to the pathological changes that contribute to SNI‐induced NP. However, it is still unclear whether lncRNA LOC100911498 interacts with a protein target in the pathophysiological progression of NP at present.

Consistent with the results that the threshold values of mechanical pain sensitivity were markedly upregulated in SNI rats, it demonstrated that abnormal pain‐like behaviors are increased in rats following SNI‐induced neuropathic injury. Moreover, our findings suggested it was siRNA silencing of LOC100911498 rather than scrambled siRNA that exert pain hypersensitivity relief. Hence, we supposed suppressing LOC100911498 treatment has inhibitory effects on mechanical hyperalgesia in SNI rats.

To further investigate what factor contributed to the mechanism through which LOC100911498 siRNA acts on NP, we used CatRAPID algorithm to identify related factors. Several P2X receptor subtypes including P2X4, P2X3, and P2X7 have been predicted to be highly correlated with lncRNA LOC100911498. The P2X4 receptor is abundantly distributed on the surface of activated microglial cells (Ulmann et al., 2008), it is not only a member of the P2X family, but also one of the key receptors promoting NP procession caused by injury to peripheral nerves (Beggs et al., 2012; Inoue & Tsuda, 2012; Tsuda et al., 2013). P2X4Rs‐mediated pain hypersensitivity has been reported in SNI rat model (Zhou et al., 2014). Thus, the mechanical hyperalgesia might be accompanied with the upregulation of the P2X4Rs in the spinal cords of SNI rats. Our data also showed that after SNI rats were treated with LOC100911498 siRNA, both P2X4 mRNA and protein expressions were reduced compared with the SNI rats, indicating LOC100911498 siRNA could block the expression of P2X4Rs and reverse the established tactile allodynia to relieve NP.

Previous records have shown that microglia in the spinal cords play a crucial role in chronic pain (Ji et al., 2013), inflammatory pain following tissue injury (Old et al., 2015), cancer pain (Zhang et al., 2005), NP following nerve injury, and spinal cord injury (Raghavendra et al., 2003), the microglia were activated and exhibit increased expression of P2X4Rs (Beggs et al., 2012; Inoue & Tsuda, 2012; Trang & BEGGS S et al., 2009; Tsuda et al., 2013). It is well known that Iba‐1 protein is expressed in activated microglia but not in unactivated microglia (Leinders et al., 2013; Tischer et al., 2016), we used Iba‐1 to identify microglia activation in the spinal dorsal horn. Our findings via double‐labeled immunofluorescence assay presented that the degree to which microglial coexpression of the P2X4Rs and Iba‐1 varied among different groups, which illustrated that on the one hand, the P2X4Rs in spinal dorsal horn microglia were activated in NP, on the other hand, LOC100911498 siRNA may suppress the activation of P2X4Rs in microglia.

Adenosine‐5′‐triphosphate released from nerve endings can activate the P2X4Rs on the microglial cells in the spinal dorsal horn (Beggs et al., 2012; Inoue & Tsuda, 2012). In addition, upregulation of P2X4Rs expression triggers the activation of the p38‐MAPK pathway, resulting in p38 converting to phosphorylated state (p‐p38; Gong et al., 2009). Meanwhile, P2X4Rs‐mediated synthesis and release of BDNF from activated microglia in vivo are dependent on calcium and p‐p38‐MAPK Trang & BEGGS S et al., 2009; Ulmann et al., 2008), all of these proteins are essential to exacerbate NP. Hence, it seems plausible that preventing the P2X4Rs‐p‐p38‐MAPK‐BDNF pathway may decrease the pain hypersensitivity in the status of NP. We found both of p‐p38 and BDNF protein levels in SNI rats are higher than the control group. After the administration of LOC100911498 siRNA in SNI rats, the high ratio of p‐p38MAPK to p38MAPK and BDNF expression was decreased versus the SNI and SNI + NCsi groups, with no difference between the SNI and SNI + NCsi groups. Additionally, the behavioral changes in the SNI rats were echoed by the significant downregulation of P2X4Rs, p‐p38, and BDNF. It is highly likely that LOC100911498 siRNA treatment reduced the upregulation of P2X4Rs and then lowered the phosphorylation of p38MAPK and BDNF expression. Taken together, our results further confirmed that LOC100911498 might be participated in the development of NP, LOC100911498 siRNA could block P2X4Rs‐mediated p38MAPK activation and BDNF release in the spinal cords, and attenuate allodynic effects.

In view of virus‐mediated gene treatment has been applied in clinical trials, RNA silencing may open a new pathway for NP management. Thence, our study not only provide a new source of biomarker to identify and monitor patients with NP, but also open the door to the underlying molecular mechanisms of the disease and lead to novel avenues to develop new analgesics with higher efficacy and fewer side effects. However, our current investigation on LOC100911498 is at an early stage, how that exerts on P2X4Rs, if LOC100911498 siRNA could regulate NP by knocking down its expression in DRGs, these points are not clear yet, which remains several challenges for the conversion of preclinical findings. Future functional and mechanistic studies on the role of LOC100911498 in NP are warranted, and the clinical efficacy and risks related with the treatment of lncRNAs also require systematic evaluation through rigorous testing.

## 5 CONCLUSION

From the above discussion, the conclusion can be displayed that LOC100911498 siRNA knockdown may alleviate NP via suppressing mutual interaction of LOC100911498 and the pain‐evoking P2X4Rs in SNI rats. Our study may provide new insight into the underlying mechanisms and may also highlight the potential of LOC100911498 as a novel therapeutic target for NP.

## CONFLICT OF INTERESTS

None declared.

## AUTHOR CONTRIBUTIONS

W.X.T conceived the project. W.X.T and L.F.Z performed the SNI model, mechanical paw withdrawal, real‐time PCR, and Western blot. Z.S.L analyzed the data and prepared figures. W.X.T and L.F.Z wrote and edited the manuscript, W.X.T revised it and supervised all experiments. All authors read and approved the final manuscript.

## ETHICAL APPROVAL AND CONSENT TO PARTICIPATE

All procedures for animal usage were complied with the guidelines of the National Institutes of Health.

### Peer Review

The peer review history for this article is available at https://publons.com/publon/10.1002/brb3.1966.

## Data Availability

The research article data used to support the findings of this study are included within the article.
